# MicroRNA-196b Regulates the Homeobox B7-Vascular Endothelial Growth Factor Axis in Cervical Cancer

**DOI:** 10.1371/journal.pone.0067846

**Published:** 2013-07-04

**Authors:** Christine How, Angela B. Y. Hui, Nehad M. Alajez, Wei Shi, Paul C. Boutros, Blaise A. Clarke, Rui Yan, Melania Pintilie, Anthony Fyles, David W. Hedley, Richard P. Hill, Michael Milosevic, Fei-Fei Liu

**Affiliations:** 1 Ontario Cancer Institute, Princess Margaret Hospital, University Health Network, Toronto, Ontario, Canada; 2 Department of Medical Biophysics, University of Toronto, Toronto, Ontario, Canada; 3 Department of Anatomy, Stem Cell Unit, College of Medicine, King Saud University, Riyadh, Saudi Arabia; 4 Informatics and Biocomputing Platform, Ontario Institute for Cancer Research, Toronto, Ontario, Canada; 5 Department of Pathology, University Health Network, Toronto, Ontario, Canada; 6 Division of Biostatistics, Princess Margaret Hospital, University Health Network, Toronto, Ontario, Canada; 7 Division of Medical Oncology, Princess Margaret Hospital, University Health Network, Toronto, Ontario, Canada; 8 Department of Radiation Oncology, Princess Margaret Hospital, University Health Network, Toronto, Ontario, Canada; 9 Department of Radiation Oncology, University of Toronto, Toronto, Ontario, Canada; Philipps University, Germany

## Abstract

The down-regulation of microRNA-196b (miR-196b) has been reported, but its contribution to cervical cancer progression remains to be investigated. In this study, we first demonstrated that miR-196b down-regulation was significantly associated with worse disease-free survival (DFS) for cervical cancer patients treated with combined chemo-radiation. Secondly, using a tri-modal approach for target identification, we determined that homeobox-B7 (HOXB7) was a *bona fide* target for miR-196b, and in turn, vascular endothelial growth factor (VEGF) was a downstream transcript regulated by HOXB7. Reconstitution of miR-196b expression by transient transfection resulted in reduced cell growth, clonogenicity, migration and invasion *in vitro*, as well as reduced tumor angiogenesis and tumor cell proliferation *in vivo*. Concordantly, siRNA knockdown of HOXB7 or VEGF phenocopied the biological effects of miR-196b over-expression. Our findings have demonstrated that the miR-196b/HOXB7/VEGF pathway plays an important role in cervical cancer progression; hence targeting this pathway could be a promising therapeutic strategy for the future management of this disease.

## Introduction

Worldwide, cervical cancer is the third most frequently diagnosed malignancy and the fourth leading cause of cancer mortality in women, with an estimated 530,000 new cases and 275,000 deaths each year [1]. Although cervical cancer incidence and mortality rates have declined over the past thirty years in the United States [2], the 5-year survival rate has remained below 40% for patients diagnosed with Stage III or IV disease [3]. Novel insights are required to better understand the mechanisms that contribute to disease progression, in order to design improved therapies for patients with locally advanced cervical cancer.

Micro-RNAs (miRNAs) are short, non-coding RNAs that regulate gene expression post-transcriptionally [4], [5], and aberrant miRNA expression has been shown to be important in many human malignancies [6]. Gene targets that contribute to tumor progression have been described for several miRNAs [7], [8], [9]; however, the biological function of the majority of miRNAs still remains unknown. One of the major challenges to miRNA target identification is the ability of miRNAs to bind mRNA targets with imperfect complementarity; hence a single miRNA can potentially regulate several hundreds or thousands of genes [10]. Unfortunately, the currently available *in silico* miRNA target prediction algorithms have high false-discovery and false-negative rates [11], [12]; thereby mandating experimental validation of miRNA targets.

Down-regulation of miR-196b in cervical cancer has been previously reported [13], but its role in tumor progression in this disease has not been previously investigated. Herein, we report down-regulation of miR-196b in primary human cervical cancer tissues and cell lines. Furthermore, we identified the HOXB7 transcription factor as a novel, direct and specific target of miR-196b, which in turn, regulates VEGF in cervical cancer. Most importantly, miR-196b down-regulation was associated with worse DFS in patients treated with chemo-radiation, highlighting the biological importance of miR-196b in cervical cancer progression.

## Materials and Methods

### Ethics Statement

Written informed consent was obtained from patients, according to a protocol approved for this study by the University Health Network Research Ethics Board. Experiments with animals were carried out in strict accordance with the protocol approved by the Animal Care Committee (ACC) of the Ontario Cancer Institute, University Health Network (Animal Use Protocol: 342.18).

### Cell Lines and Transfections

Human cervical cancer cell lines (ME-180, SiHa, and HT-3) were obtained from American Type Culture Collection (ATTC), and grown in α-MEM supplemented with 10% FBS at 37°C, 5% CO_2_. All cells were authenticated every six months at the Centre for Applied Genomics (Hospital for Sick Children, Toronto, Canada) using the AmpF/STR Identifier PCR Amplification Kit (Applied Biosystems), and determined to be free from *Mycoplasma* contamination using the MycoAlert Mycoplasma Detection Kit (Lonza). ME-180 and SiHa cells were transfected using the LipofectAMINE 2000 (Invitrogen) forward transfection protocol, according to the manufacturer’s instructions. Pre-miR Negative Control #1 (NC), Pre-miR-196b (Ambion), All Stars Negative Control (siNEG), siHOXB7 and siVEGF (Qiagen) were all transfected at a final concentration of 30 nmol/L.

### miRNA Expression Profiling of Cell Lines

Total RNA was isolated from SiHa, ME-180 and HT3 cervical cancer cell lines using the mirVana miRNA Isolation Kit (Ambion) according to the manufacturer’s instructions. FirstChoice® Total RNA: Human Normal Cervix Tissue (Ambion) from 3 different tissue donors was utilized as normal comparators. Expression levels of 377 miRNAs and 3 snoRNAs (controls) were assayed in the cervical cancer cell lines and normal cervix tissues using the TaqMan® Low Density Array (TLDA) Human MicroRNA Panel (Applied Biosystems), with the Applied Biosystems 7900HT Real-Time PCR System, as we have previously described [14].

### miRNA Expression Profiling of Patient Tissues

Flash-frozen punch biopsies were obtained from patients with locally advanced cervical cancer who were planned to receive primary treatment with standard chemo-radiation, consisting of external-beam radiotherapy to the primary cervical tumor and pelvic lymph nodes (45 to 50 Gy total, in 1.8-to-2-Gy daily fractions with 18-to-25-MV photons), combined with weekly doses of cisplatin (40 mg/m^2^ total, 5 doses). FIGO (International Federation of Gynecologists and Obstetricians) staging was determined using a combination of: pretreatment evaluation under anesthesia, computed tomography (CT) scans of the abdomen and pelvis, chest x-ray, and magnetic resonance imaging (MRI) of the pelvis. MRI was also used to determine lymph node status; pelvic and para-aortic lymph nodes were classified as positive for metastatic disease if the MRI short-axis dimension was >1 cm and equivocal if it was 8 to 10 mm. After biopsy, the specimens were placed in optimal cutting temperature (OCT) storage medium for histopathologic examination, then flash-frozen in liquid nitrogen. H&E-stained tissue sections were cut from the OCT-embedded material and evaluated by a gynecologic pathologist (B Clarke). Total cell content (stroma and tumor cells) was estimated for all tissue samples using a light microscope, and only samples containing >70% tumor cells were considered for further analysis (n = 79). The clinical characteristics of these 79 patients are provided in Table 1. The median follow-up time for this cohort was 3 years. Flash-frozen normal cervix tissues obtained from 11 patients who underwent hysterectomy for benign causes served as normal comparators.

**Table 1:** Clinical parameters of 79 cervical cancer patients.

Category	Subcategory	N = 79	Percent
Age, years	Median	48	
	Range	26–84	
Tumour size	≤5 cm	48	61%
	>5 cm	31	39%
FIGO stage	IB	24	30%
	IIA	2	3%
	IIB	35	44%
	IIIA	0	0%
	IIIB	18	23%
Histology	Squamous	74	94%
	Adenocarcinoma	4	5%
	Adenosquamous	1	1%
Pelvic node involvement	Positive	25	32%
	Equivocal	15	19%
	Negative	39	49%

Two sections of 50-micron thickness were cut from the OCT-embedded flash-frozen tissues and placed in a nuclease-free microtube. Total RNA was isolated using the Norgen Total RNA Purification Kit (Norgen Biotek), according to the manufacturer’s instructions. Global miRNA expression was measured in the cervical cancer and normal cervix tissues with the TaqMan® Low Density Array (TLDA) Human MicroRNA A Array v2.0 (Applied Biosystems) using the Applied Biosystems 7900HT Real-Time PCR System, as already described [14].

### Quantitative Real-time PCR Analysis of miRNAs and mRNAs

Total RNA was isolated from the cell lines using the Total RNA Purification Kit (Norgen Biotek), according to the manufacturer’s instructions. The expression of miR-196b was measured by quantitative real-time polymerase chain reaction (qRT-PCR) using the standard TaqMan MicroRNA Assay (Applied Biosystems). Briefly, RNA was first reverse transcribed using the TaqMan MicroRNA Reverse Transcription (RT) Kit and a stem-loop primer specific for miR-196b (Applied Biosystems) [15]. The 2^-ΔΔCt^ method was used to calculate relative levels of miR-196b expression, using RNU44 as a reference gene [16].

The RT products were amplified with miR-196b-specific primers, as we previously described [14]. The expression levels of previously-described (c-myc, BCL2, HOXA9, MEIS1), and candidate mRNA targets for miR-196b (ANKHD1, CTDSP2, FGFR1, HDAC, HOXA7, HOXB7, KRT8, PUM2, SLC9A6, SMC3, SMG7, SR140), and HOXB7 (VEGF, Ku70, Ku80, DNA-PK, FGF2, MMP2, WNT5a, PDGFA, THBS2) were also measured by qRT-PCR. One microgram of total RNA was reverse-transcribed using SuperScript II Reverse Transcriptase (Invitrogen) according to the manufacturer’s instructions. Quantitative RT-PCR was performed using SYBR Green PCR Master Mix (Applied Biosystems) and PCR primers (Table S1) designed using Primer 3 Input. The 2^-ΔΔCt^ method was used to calculate relative levels of gene expression, with GAPDH as a reference gene [16].

### Cell Viability, Proliferation and Colony-Forming Assays

The viability of transfected cells was assessed by the Trypan blue exclusion assay. ME-180 and SiHa cells were transfected in triplicate with 30 nmol/L of pre-miR-196b, NC, siHOXB7, siVEGF, or siNEG and incubated at 37°C, 5% CO_2_. At 48 and 72 hours post-transfection, cells were trypsinized, stained with Trypan blue and counted using a hemocytometer. Cell proliferation was examined using the CellTiter 96 Non-Radioactive Cell Proliferation Assay (MTS Assay) (Promega BioSciences), according to the manufacturer’s instructions. For colony formation assays, cells were transfected with 30 nmol/L of pre-miR-196b, Pre-miR Negative Control #1, siHOXB7, siVEGF, or siNEG and incubated at 37°C, 5% CO_2_. At 48 hours post-transfection, cells were re-seeded at low density in 6-well plates in triplicate. Cells were incubated at 37°C, 5% CO_2_ for 10–12 days, then fixed and stained with 0.1% crystal violet in 50% methanol. The number of colonies containing at least 50 cells was counted, and the surviving fraction was calculated by comparison with cells transfected with Negative Control.

### Cell Migration and Invasion Assays

BD BioCoat Matrigel Invasion Chambers and Control Inserts (BD Biosciences) were used to assay migration and invasion of transfected cells. Chambers contained a polyethylene terephthalate membrane with 8 µm pores. ME-180 and SiHa cells were transfected with 30 nmol/L of pre-miR-196b, NC, siHOXB7, siVEGF, or siNEG and incubated at 37°C, 5% CO_2_. At 24 hours after transfection, 1.5×10^5^ cells were re-seeded inside each chamber with medium containing low serum (1% FBS). The chambers were placed in a 24-well plate, with high serum (20% FBS) medium in each lower chamber to serve as a chemo-attractant. Cells were incubated at 37°C, 5% CO_2_ for 48 hours, then the membranes were washed, stained, and mounted onto slides. A light microscope was used to count the number of migrating or invading cells. Relative migration was calculated by comparison with cells transfected with the negative control. Percent invasion was calculated as the number of cells that invaded through the Matrigel insert, divided by the number of cells that migrated through the control insert.

### Cell Cycle Analysis

Cell cycle analysis was performed on ME-180 and SiHa cells after transfection with 30 nmol/L of pre-miR-196b or NC, to measure the fraction of cells in the sub-G_1_ phase of the cell cycle. Cells were harvested and washed twice in FACS buffer (PBS/0.5% BSA), re-suspended in 1 mL of FACS buffer, then fixed in 1 mL of ice-cold 70% ethanol. After 1 h of incubation on ice, cells were washed again and re-suspended in 500 uL of FACS buffer containing 40 µg/mL RNase A (Sigma) and 50 µg/mL propidium iodide, then incubated in the dark at room temperature for 30 minutes. Cells were analyzed in the BD FACScalibur (Becton Dickinson) using the FL-2A and FL-2W channels. The flow cytometry data were analyzed using FlowJo 7.5 software (Tree Star).

### In vivo Experiments

Six- to 8-week-old severe combined immunodeficient (SCID) female mice were utilized for xenograft experiments, according to guidelines of the Animal Care Committee, Ontario Cancer Institute, University Health Network. Cells were transfected with pre-miR-196b or NC and incubated at 37°C, 5% CO_2_. At 48 hours post-transfection, cells were harvested and 5×10^5^ viable cells were diluted in 100 µL of growth medium. Cells were injected intramuscularly into the left gastrocnemius muscle of female SCID mice. Tumor plus leg diameter was measured twice a week and mice were euthanized when 15 mm was attained. Tumors were removed at 25 days after implantation and immediately fixed in 10% buffered formalin for 24 h, placed in 70% ethanol for 24 h, embedded in paraffin, and sectioned (5 µM) for immunostaining. In addition to hematoxylin and eosin (H&E) staining, cluster of differentiation 31 (CD31) was used for assessing tumor angiogenesis, Ki-67 for tumor cell proliferation, and terminal deoxynucleotidyl transferase-mediated dUTP nick end labeling (TUNEL) for apoptosis.

### Tri-modal Approach for miRNA Target Identification

Candidate mRNA targets of miR-196b were determined using a previously-described tri-modal approach [8] combining: i) all predicted targets of miR-196b from five *in silico* miRNA target prediction databases (TargetScan, PicTar, GenMir^++^, miRBase, miRDB); ii) mRNAs up-regulated at least 2-fold in cervical cancer tissues compared to normal cervix tissues, using two independent publicly-available microarray datasets [17], [18]; and iii) mRNAs down-regulated at least 0.5-fold at both 24 and 72 hours after transfection with 30 nmol/L of pre-miR-196b, where transcript levels were measured using the Whole Human Genome 4×44 K One-Color Array (Agilent).

### Luciferase Reporter Assay

Wildtype or mutant fragments of the 3′-untranslated region (UTR) of HOXB7 containing the predicted binding site (position 220–226) for miR-196b were individually amplified by AmpliTaq Gold DNA Polymerase (Applied Biosystems) using the primers listed in Table S1. The PCR products were purified, then cloned downstream of the firefly *luciferase* gene in the pMIR-REPORT vector (Ambion) at the *Spe*I and *Hind*III restriction sites, to produce the pMIR-HOXB7 or pMIR-HOXB7-mut vector. ME-180 and SiHa cells were co-transfected with 100 nmol/L of pre-miR-196b or NC, and 100 ng of the reporter vector of interest. As a reference control, 50 ng of pRL-SV vector (Promega) containing the *Renilla luciferase* gene was also transfected with each condition. Firefly and renilla luciferase activities were measured at 24 hours post-transfection using the Dual-Glo Luciferase Assay System (Promega) according to the manufacturer’s instructions.

### Immunoblotting

Cells were transfected with either 100 nmol/L of pre-miR-196b or NC, and total protein extracts were harvested on ice after 48 and 72 hours. Proteins of interest were probed with rabbit anti-HOXB7 (1∶500 dilution; Invitrogen) or mouse anti-GAPDH (1∶10,000 dilution; Sigma), and detected with IRDye fluorescent secondary antibodies (1∶20,000 dilution, LI-COR). GAPDH was used as a loading control. Immunoblots were scanned and quantified using the Odyssey Infrared Imaging System (LI-COR).

### Enzyme-linked Immunosorbent Assay (ELISA)

Cells were transfected with 30 nmol/L of siHOXB7 or siNEG, and the level of secreted VEGF was measured at 48 and 72 hours post-transfection, using the Human VEGF DuoSet ELISA (R&D Systems) according to manufacturer’s instructions.

### 5-aza-2′-deoxycytidine Treatment

ME-180 and SiHa and were seeded in 24-well plates and incubated overnight at 37°C, 5% CO_2_. Media containing 2 µM 5-aza-2′-deoxycytidine (5-aza-DCT) (Sigma-Aldrich) was added to cells, 1 and 3 days after seeding, respectively. Cells were harvested 4 days after seeding and total RNA was isolated for qRT-PCR analysis of miR-196b expression.

### Statistical Analysis

All experiments have been performed at least three independent times, and the data are presented as the mean ± standard error of the mean (SEM). The Student’s t-test function (unpaired, two-tailed) in Microsoft Excel (Microsoft, Redmond, WA) was used to compare two treatment groups. Graphs were plotted using GraphPad Prism software (GraphPad software, San Diego, CA). The Kaplan-Meier method was used for univariate analysis, and the log-rank test was used to examine associations between miR-19b expression and DFS, where the expression was dichotomized at the median value. The relationship between tumor size and miR-196b expression was investigated using Pearson’s correlation. Correlations between miR-196b expression with either FIGO stage or nodal status were analyzed using one-way ANOVA.

## Results

### miR-196b was Significantly Down-regulated in Primary Cervical Cancer Tissues and Cell Lines, and was Strongly Associated with DFS

**Figure 1:**
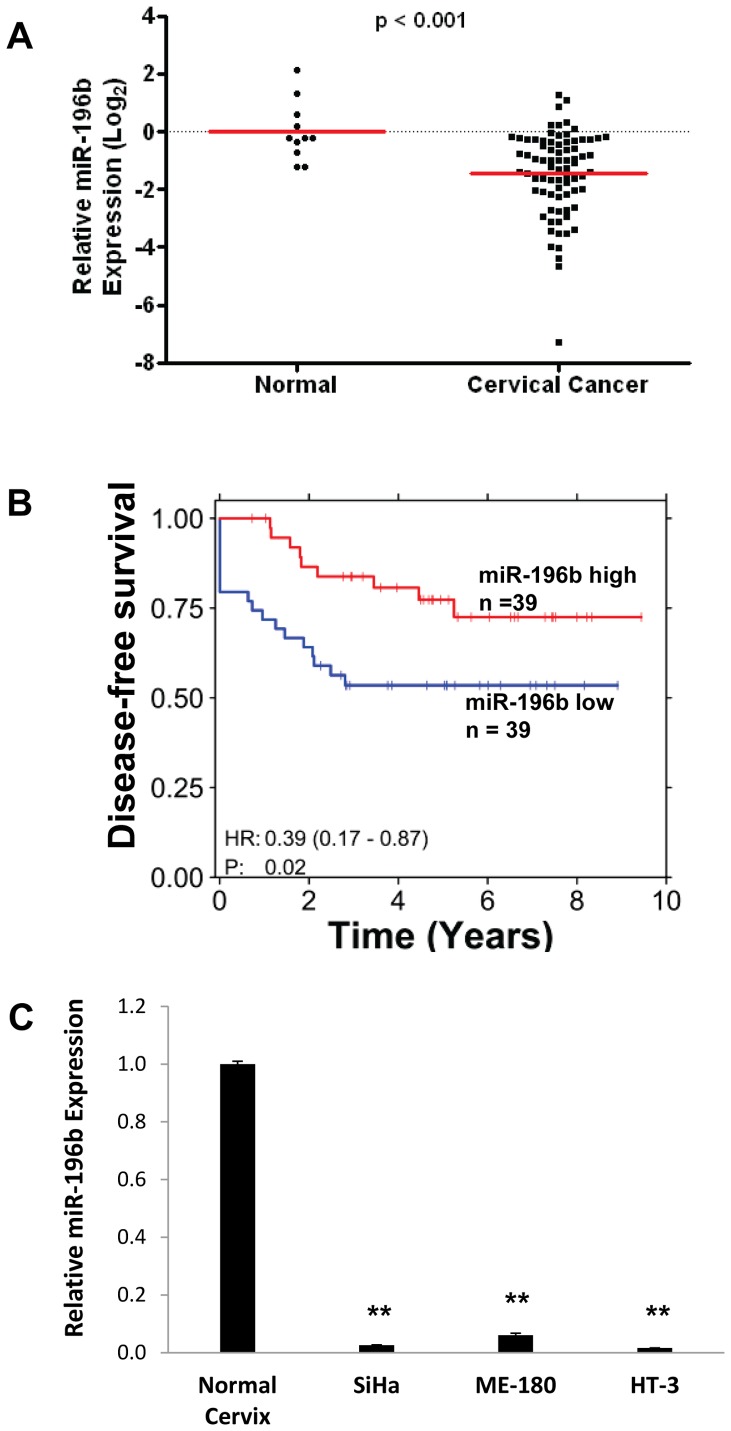
miR-196b down-regulation in cervical cancer. **A)** miR-196b expression levels were measured using qRT-PCR in 79 primary cervical cancer samples, compared to 11 normal cervix epithelial tissue controls. **B)** Kaplan-Meier analysis of DFS in patients with cervical cancer. Red, patients with higher than median miR-196b expression level (n = 39); blue, patients with lower than median miR-196b expression (n = 39); one patient was removed from survival analysis due to missing survival information. **C)** Basal levels of miR-196b in three cervical cancer cell lines (SiHa, ME-180, and HT-3), as compared to normal cervix epithelial tissues, assayed by qRT-PCR. **P<0.01.

Expression of miR-196b was significantly reduced by almost 4-fold in 79 primary cervical cancer tissues compared to 11 normal cervix tissues (*P*<0.001; Fig. 1A). Importantly, patients with lower than median miR-196b expression level at the time of diagnosis experienced worse DFS compared to those with higher miR-196b expression (*P* = 0.02; hazard ratio = 0.39; Fig. 1B). miR-196b expression was not significantly correlated with tumor size (*P* = 0.12), FIGO stage (*P* = 0.14), or nodal status (*P* = 0.60). Global miRNA expression profiling conducted on three cervical cancer cell lines (ME-180, SiHa and HT-3) also confirmed the down-regulation of miR-196b in cervical cancer. From the 55 miRNAs that were deregulated at least 2-fold in all three cell lines compared to 3 normal cervix tissues, miR-196b was amongst the most significantly down-regulated miRNAs (Fig. 1C), consistent with a previously published miRNA expression profiling study [13].

To explore whether miR-196b down-regulation was epigenetically determined, in addition to chromosomal loss [19], ME-180 and SiHa cells were treated with the demethylating agent 5-aza-2′-deoxycytidine (5-aza-DCT). This treatment resulted in only a minimal increase in miR-196b expression, indicating that promoter methylation was unlikely to be a major mechanism for miR-196b under-expression (Fig. S1A). Furthermore, examination of publically-available microarray datasets of gene expression in primary cervical cancer tissues did not reveal any significant alterations in the expression of DICER, drosha, DGCR8, Exportin-5, or any subunits of RNA Polymerase II, which are all involved in miRNA biogenesis and processing (data not shown).

### miR-196b Over-expression Significantly Reduced Cell Viability, Clonogenicity, Proliferation and Invasion

**Figure 2:**
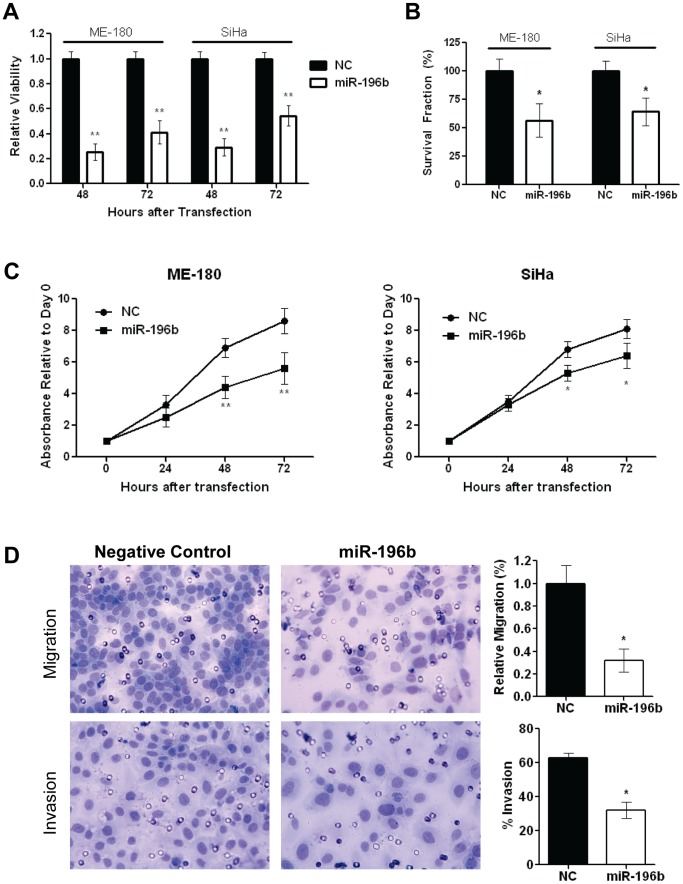
*In vitro e*ffects of miR-196b over-expression. **A)** Relative viability of ME-180 and SiHa cells were assessed at 48 and 72 hours post-transfection with pre-miR-196b (30 nmol/L), compared to Negative Control pre-miR (30 nmol/L), using the Trypan Blue assay. **B)** Clonogenicity of ME-180 and SiHa cells were assessed by transfection with 30 nmol/L of pre-miR 196b or Negative Control pre-miR. At 48 hours post-transfection, cells were harvested then counted and re-seeded at low density in 6-well plates. After 10 days of incubation, cells were fixed and stained and the number of colonies (>50 cells) were counted. **C)** Relative proliferation of ME-180 and SiHa cells were examined at 24, 48, and 72 hours post-transfection with pre-miR-196b (30 nmol/L), compared to Negative Control pre-miR (30 nmol/L), using the MTS assay. **D)** Representative images (left) and histograms (right) depicting migratory ability (top) and invasiveness (bottom) of ME-180 cells that were transfected with 30 nmol/L of pre-miR 196b or Negative Control pre-miR, harvested at 24 hours post-transfection, then counted and re-seeded in the invasion chambers. Cell migration (top), and invasion (bottom), assessed at 48 hours after seeding in transwell chambers. All data represent the mean ± SEM from 3 independent experiments. NC, pre-miR Negative Control; *P<0.05; **P<0.01.

To assess the biological significance of miR-196b down-regulation, cells were transfected with 30 nmol/L NC or pre-miR-196b. Up-regulation of miR-196b expression was sustained for up to 72 hours after transfection (Fig. S1B). Transfection with pre-miR-196b led to significantly decreased cell viability compared to controls at 48 and 72 hours post-transfection (ME-180∶25% at 48 h; 41% at 72 h, SiHa: 29% at 48 h; 54% at 72 h) (Fig. 2A). In addition, miR-196b over expression resulted in significant reductions in clonogenicity (ME-180∶57% compared to NC, SiHa: 64% compared to NC) (Fig. 2B), proliferation (ME-180∶36% at 48 h, 35% at 72 h, SiHa: 22% at 48 h; 21% at 72 h) (Fig. 2C), and migration (32% compared to NC), plus invasion (32% *vs*. 63% for NC) (Fig. 2D). Cells treated with pre-miR-196b demonstrated a small, but not statistically significant, increase in the percentage of cells in the sub G_1_ population, accompanied by a small decrease in the G_0_–G_1_ population (Fig. S1C).

### miR-196b Over Expression Suppressed Tumor Angiogenesis and Tumor Cell Proliferation in vivo

Cells transfected with pre-miR-196b did not show a statistically significant difference in tumor growth in mice, compared to NC-treated cells (Fig. S2). At 25 days post-implantation however, CD31 immunostaining was nonetheless observed to be reduced to 69% in pre-miR-196b-treated tumors compared to control tumors (Fig. S3, top). Furthermore, this was associated with a modest yet significant reduction in Ki-67 expression (46% *vs*. 53% for cells treated with NC) (Fig. S3, middle), and a minor, but not statistically significant increase in TUNEL staining (Fig. S3, bottom). Hence, our data suggested that pre-miR-196b contributed to reduced angiogenesis and tumor cell proliferation.

### miR-196b Directly Targets HOXB7

The down-regulation of miR-196b in cervical cancer tissues and cell lines, and the significant phenotypic effects of miR-196b over-expression both *in vitro* and *in vivo*, indicated that miR-196b appears to be an important mediator of cervical cancer progression. Thus, a tri-modal strategy [8] was utilized to identify potential mRNA targets that could account for these phenotypic changes (Fig. S4). This method identified 15 overlapping candidate targets (ANKHD1, CALM3, CLK2, CTDSP2, FGFR1, HDAC, HOXA7, HOXB7, KRT8, PCCB, PUM2, SLC9A6, SMC3, SMG7, SR140). For target validation, ME-180 cells were transfected with pre-miR-196b or NC, and transcript levels at 24 hours post-transfection were measured with qRT-PCR for 12 candidate targets (suitable primers could not be designed for the other 3 transcripts), plus 4 previously-described targets of miR-196b (c-myc, BCL2, HOXA9, MEIS1). None of the previously-described targets were significantly altered after miR-196b over-expression (Fig S5A). Only 5 of the 12 tested candidate targets were significantly down-regulated after miR-196b over-expression (Fig. S5B); HOXB7 was selected for further functional evaluation since it was the candidate target that demonstrated the greatest level of down-regulation (40% compared to controls). Furthermore, HOXB7 is a member of the *Hox* gene cluster, a family of genes which have been reported to be dysregulated in various malignancies [20], and the miR-196 family is known to target the mammalian *Hox* genes [21].

**Figure 3:**
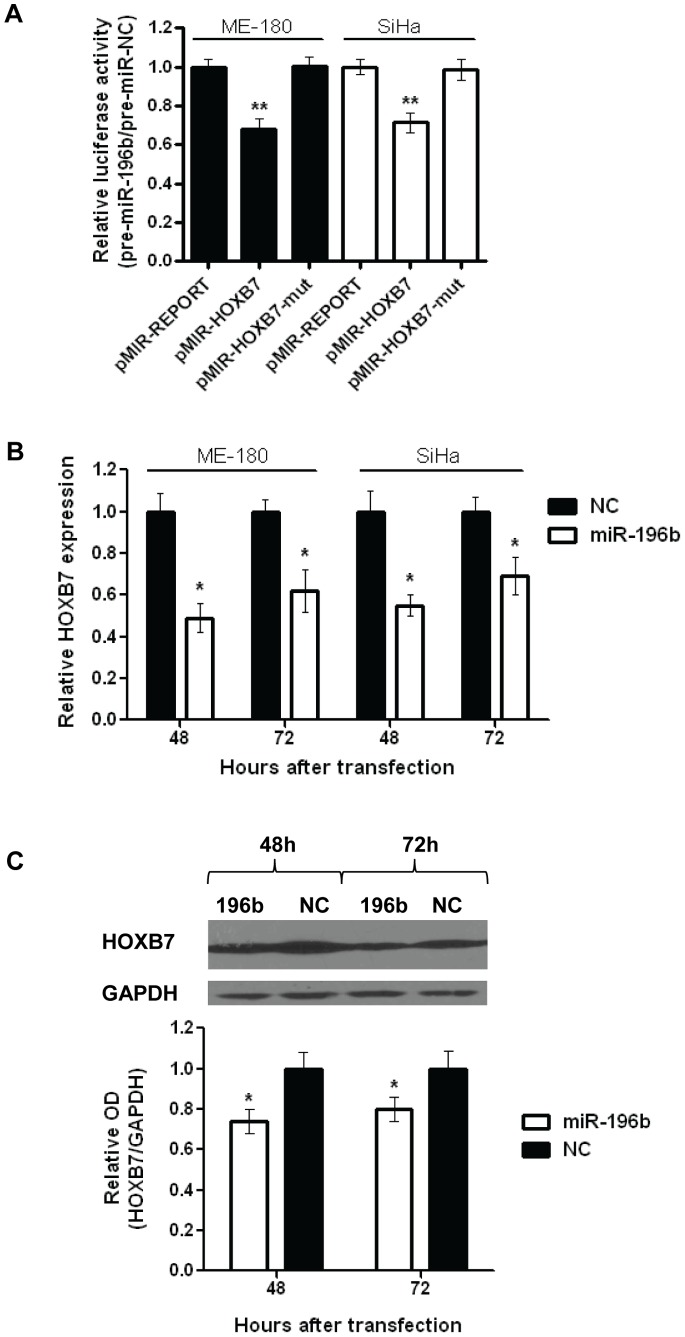
Identification of HOXB7 as an mRNA target of miR-196b. **A)** Relative luciferase activity of ME-180 or SiHa cells at 24 hours after co-transfection with pMIR-REPORT, pMIR-HOXB7 or pMIR-HOXB7-mut vectors and pre-miR-196b or NC (30 nmol/L). **B)** Relative HOXB7 mRNA expression levels in ME-180 or SiHa cells after transfection (48, and 72 hrs) with pre-miR-196b or NC (30 nmol/L), as measured by qRT-PCR. Expression levels were normalized to GAPDH expression. **C)** Representative Western blot image (top), and relative quantification of HOXB7 protein levels (bottom) after transfection (48, and 72 hrs) with pre-miR-196b or NC (30 nmol/L). All data represented the mean ± SEM from 3 independent experiments. OD, optical density; NC, pre-miR Negative Control; *P<0.05; **P<0.01.

A luciferase binding assay confirmed that miR-196b directly and specifically interacted with the 3′-UTR of HOXB7. In comparison to control cells transfected with pMIR-REPORT, cells transfected with pMIR-HOXB7 showed reduced luciferase activity (ME-180∶68%, SiHa: 71%) when co-transfected with pre-miR-196b (Fig. 3A). This inhibitory effect was completely abrogated with pMIR-HOXB7-mut, which contained a mutation in the miR-196b binding site. In addition, transfection with pre-miR-196b resulted in significantly reduced HOXB7 mRNA transcript (ME-180∶49% at 48 h, 62% at 72 h; SiHa: 55% at 48 h, 69% at 72 h) (Fig. 3B), and protein (74% at 48 h; 80% at 72 h) (Fig. 3C) levels at 48 and 72 hours post-transfection. There appeared to be a greater fold change in HOXB7 at the mRNA level compared to protein, which is not surprising since miRNAs interact directly with mRNA transcripts and not proteins. In addition, protein translation can be affected by a number of mechanisms that occur upstream, such as nuclear export of RNA transcripts and recruitment of ribosomal subunits.

### VEGF is a Relevant Downstream Target of HOXB7

**Figure 4:**
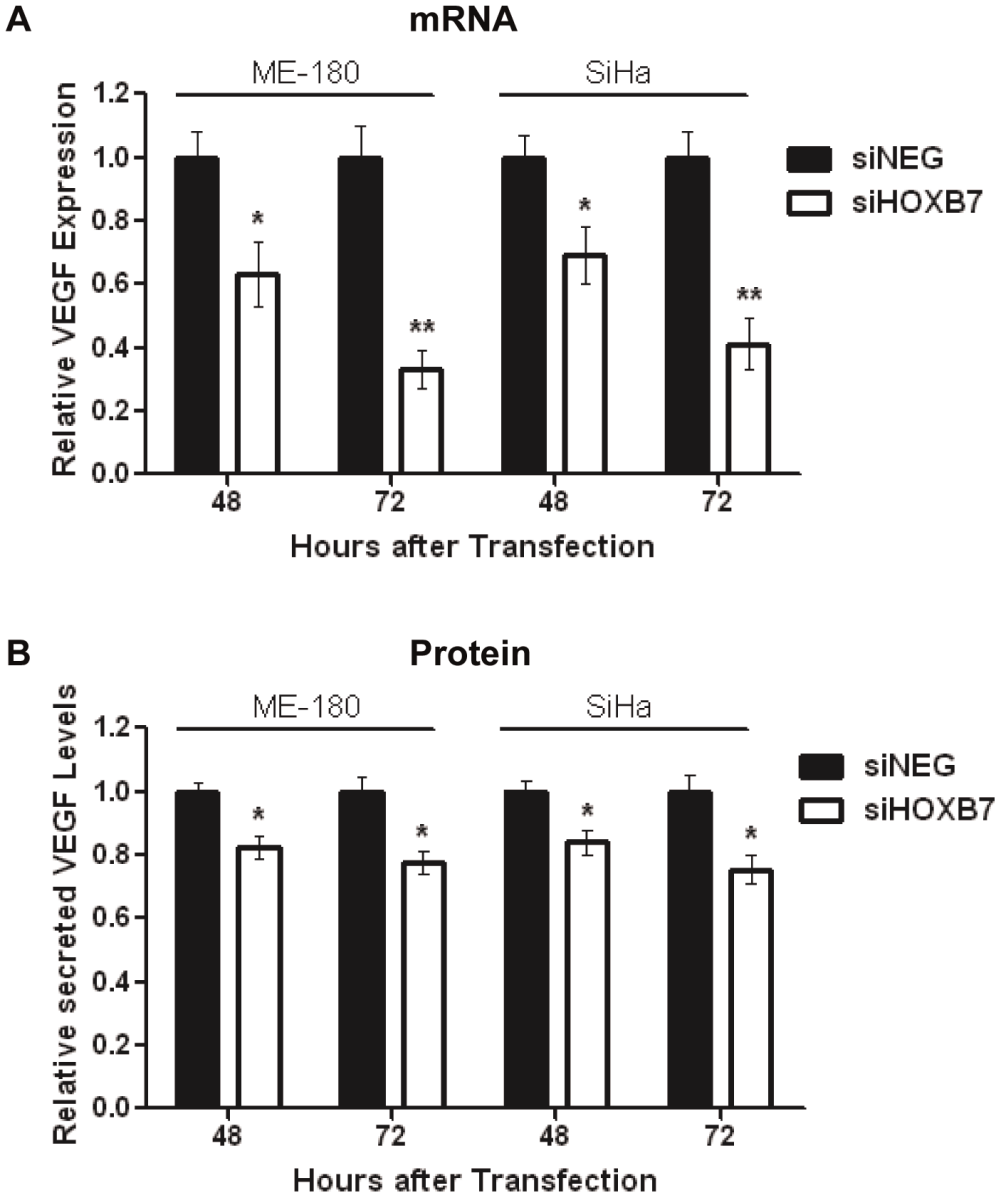
Identification of VEGF as a target of the HOXB7 transcription factor. **A)** Relative VEGF expression at the mRNA level after transfection of ME-180 or SiHa cells with siHOXB7 or siNEG (30 nmol/L), as determined by qRT-PCR. Expression levels were normalized to GAPDH expression. **B)** Relative levels of secreted VEGF protein after transfection of ME-180 or SiHa cells with siHOXB7 or siNEG (30 nmol/L), as determined by ELISA. All data represented the mean ± SEM from 3 independent experiments. siNEG, All Stars Negative Control; *P<0.05; **P<0.01.

Since HOXB7 is a transcription factor, it was necessary to determine which gene(s) regulated by HOXB7 could be relevant in this context for cervical cancer. Hence, cells were transfected with 30 nmol/L of siHOXB7 or siNEG, and mRNA levels of known HOXB7 targets such as Ku70, Ku80, DNA-PK, FGF2, MMP2, WNT5a, PDGFA, thrombospoindin 2 (THBS2) and VEGF [22], [23], [24], [25] were measured at 48 hours post-transfection. VEGF demonstrated the greatest level of down-regulation (43%) following HOXB7 knockdown (Fig. S6A). Concordantly, significant reduction in VEGF mRNA (ME-180∶63% at 48 h, 33% at 72 h; SiHa: 69% at 48 h, 41% at 72 h) (Fig. 4A) and secreted protein (ME-180∶82% at 48 h, 77% at 72 h; SiHa: 84% at 48 h, 75% at 72 h) (Fig. 4B) levels were observed at both 48 and 72 hours post-transfection with siHOXB7, confirming that VEGF was indeed a relevant downstream HOXB7 target in this disease. Furthermore, other pro-angiogenic known targets of HOXB7 (FGF2, MMP2, WNT5a, PDGFA, THBS2) were not significantly altered following HOXB7 knockdown (Fig. S6A) or miR-196b overexpression (Fig. S6E), suggesting that HOXB7 mediated angiogenesis *via* VEGF in this context.

### Knockdown of HOXB7 or VEGF Recapitulated the Biological Effects Observed Following miR-196b Over Expression

**Figure 5:**
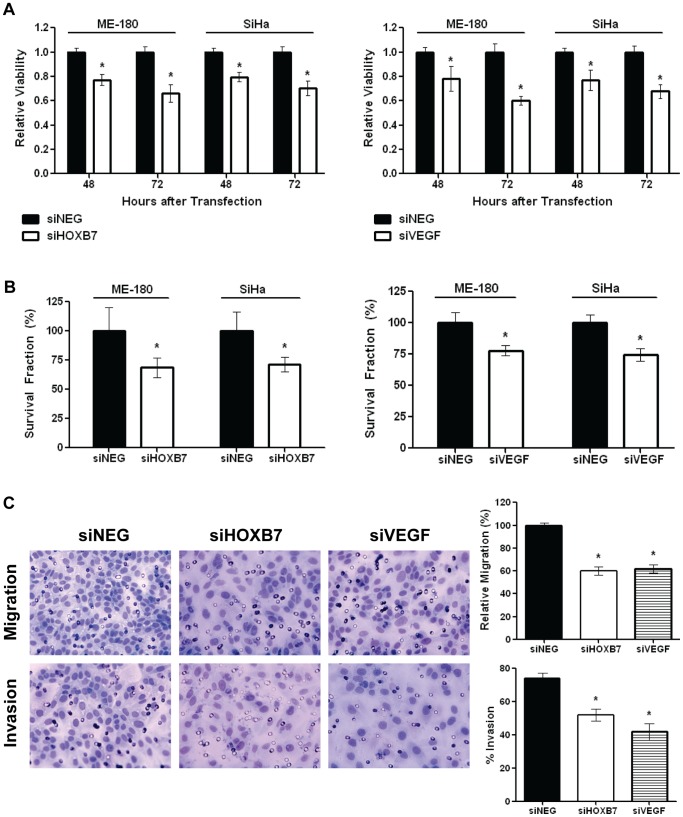
Downstream effects of siHOXB7 and siVEGF. ME-180 and SiHa cells were transfected with siHOXB7, siVEGF or siNEG (30 nmol/L): **A)** Relative cell viability assessed using the Trypan blue assay at 48 and 72 hours post-transfection with siHOXB7 (left) or siVEGF (right); **B)** Cells were harvested at 48 hours post-transfection, then counted and re-seeded at low density in 6-well plates; after 10 days of incubation, cells were fixed and stained and colonies (>50 cells) were counted. Histograms depict the relative number of surviving colonies post-transfection with siHOXB7 (left), and siVEGF (right). **C)** Representative images (left) and histograms (right) depict migratory ability (top), and invasiveness (bottom) after transfection. All data represented the mean ± SEM from 3 independent experiments. siNEG, All Stars Negative Control; *P<0.05.

To further corroborate this newly-described pathway of miR-196b targeting HOXB7 which in turn regulated VEGF, ME-180 and SiHa cells were treated with siHOXB7 or siVEGF to determine whether these interventions could recapitulate the effects of miR-196b over-expression. Knockdown of HOXB7 and VEGF transcript and protein levels were sustained for up to 72 hours after siRNA transfection (Fig. S6B, S6C, and S6D). We observed that cell viability was reduced significantly after transfection with either siHOXB7 (ME-180∶77% at 48 h, 66% at 72 h; SiHa: 80% at 48 h, 70% at 72 h) (Fig. 5A, left) or siVEGF (ME-180∶78% at 48 h, 60% at 72 h; SiHa: 77% at 48 h, 68% at 72 h) (Fig. 5A, right), similar to the effects observed after transfection with pre-miR-196b (ME-180∶25% at 48 h, 41% at 72 h; SiHa: 29% at 48 h, 54% at 72 h) (Fig. 2A). Clonogenicity decreased significantly following either siHOXB7 (ME-180∶69%; SiHa: 71%) (Fig. 5B, left) or siVEGF (ME-180∶78%; SiHa: 75%) (Fig. 5B, right) transfection, as previously observed for pre-miR-196b transfection (ME-180∶57% compared to NC; SiHa: 64% compared to NC) (Fig. 2B). Cell migration (Fig. 5C, top) was also reduced significantly following transfection with either siHOXB7 (60%) or siVEGF (62%), similar to the results observed following pre-miR-196b transfection (32% *vs.* NC) (Fig. 2D, top). Finally, cell invasion also significantly decreased after transfection with either siHOXB7 (52% *vs*. 74% for control cells) or siVEGF (42% *vs*. 74% for control cells) (Fig. 5C, bottom), all recapitulating the effects observed after transfection with pre-miR-196b (32% *vs*. 63% for control cells) (Fig. 2D, bottom).

## Discussion

In this study, we identified that miR-196b was significantly down-regulated in both cervical cancer cell lines and primary tissues, which promoted tumour cell proliferation, migration, invasion, and angiogenesis, mediated through VEGF regulation by HOXB7. MicroRNA-196b was one of the six deregulated miRNAs first reported by Lui *et al* for human cervical cancer [13]. Its biological role was first described for endometriosis [26]; since then, miR-196b has been reported to be deregulated in various human malignancies aside from cervical cancer, including over-expression in acute lymphoblastic leukemia (ALL) [27] and colon cancer [28]; as well as under-expression in glioblastoma [29] and B-cell lineage ALL [30]. These discordant observations highlight the fact that miR-196b can function as either an oncogene or a tumor suppressor, as described for many miRNAs. In glioblastoma, miR-196b levels have been positively correlated with overall survival [29]. In contrast, we report for the first time that lower levels of miR-196b were associated with worse DFS for cervical cancer, by promoting cellular proliferation, clonogenicity, migration and invasion *in vitro*, as well as tumor cell proliferation and angiogenesis *in vivo*.

The mechanisms for miR-196b down-regulation are complex. Using array comparative genomic hybridization (aCGH) profiling of cervical cancer samples, the chromosomal location of miR-196b (7p15.2) was noted to be a region with a high level of homozygous loss in squamous cell cervical carcinoma [19]; hence this might be one explanation for down-regulation of miR-196b in this disease. miR-196b has also been reported to be epigenetically regulated in gastric cancer [31], which was not corroborated based on our data (Figure S2).

Few studies have identified mRNA targets of miR-196b, aside from c-myc [30], and the *Hox* gene cluster [21]. The *Hox* gene family consists of a set of 39 genes which encode transcription factors that direct the basic structure and orientation of an organism during embryonic development [32], regulating many crucial processes such as differentiation, apoptosis, motility, angiogenesis and receptor signaling [20]. Aberrant *Hox* gene expression has been reported to mediate oncogenesis in many human cancers, including hepatocellular [33], ovarian [34], as well as acute myeloid leukemia (AML) [35]. There are at least three mechanisms that have been described which can lead to Hox gene deregulation: a) over-expression of *Hox* genes in a specific tissue type; b) epigenetic deregulation, whereby *Hox* genes are silenced in a tissue when they should normally be expressed; and c) temporo-spatial deregulation, whereby *Hox* gene expression in a tumor arising in a specific tissue temporo-spatially differs from that in normal tissue [36]. In this current study, we provide evidence for an alternate mechanism for HOXB7 deregulation, *via* miR-196b, in cervical cancer.

Our current study also demonstrated that VEGF was a downstream transcriptional target of HOXB7 in cervical cancer, which has also been reported for breast cancer [23], as well as multiple myeloma [25]. VEGF is a known key mediator of angiogenesis [37]; a major hallmark of human cancers [38]. In contrast, other pro-angiogeneic factors that are known targets of HOXB7 (FGF2, MMP2, WNT5a and PDGF) were not significantly altered following HOXB7 knockdown or miR-196b overexpression [23], [25]. Although VEGF was initially regarded to be an endothelial-specific ligand, reports have shown that VEGF can promote cancer cell proliferation [39], [40], migration and invasion [41], [42]. Interestingly, serum VEGF levels have been shown to be a prognostic marker for DFS in cervical cancer patients, whereby high pre-treatment VEGF levels were associated with worse survival [43], [44]. In addition, alterations in the serum concentration of VEGF have been used to measure treatment response in cervical cancer patients [45]. Serum expression of VEGF might indeed be a superior read-out of angiogenic activity in cervix tumors, compared to tissue expression of VEGF. Unfortunately, sera samples were not available from the patients in this current study.

A humanized anti-VEGF monoclonal antibody (A.4.6.1) that recognizes all biologically active isoforms of VEGF and prevents their binding to VEGF receptors (VEGFR-1 and VEGFR-2), Bevacizumab, has been shown to be effective in treating several human malignancies by blocking angiogenesis [46]. Thus far, Bevacizumab has been approved by the FDA for treating metastatic colorectal cancer, non-small cell lung cancer, glioblastoma, and metastatic kidney cancer. A multi-centre randomized Phase III clinical trial (GOG240) has recently been completed, in which Bevacizumab in combination with standard treatment was evaluated in cervical cancer patients with advanced (stage IVB), persistent, or recurrent cervical cancer. A National Cancer Institute (NCI) press release in February 2013 announced that the Phase III trial demonstrated that the addition of Bevacizumab significantly improved median survival by 3.7 months.

In conclusion, we report for the first time that miR-196b is a novel tumor suppressor in cervical cancer, by regulating the transcription factor HOXB7, which in turn, induced VEGF expression. The resulting phenotype of miR-196b down-regulation included increased cell growth, clonogenicity, migration and invasion, as well as increased tumor cell proliferation and vascularity *in vivo*. Furthermore, patients with lower miR-196b expression experienced a worse 5-year DFS. Hence, this novel axis of miR-196b∼HOXB7∼VEGF might well provide the biological rationale for the potential efficacy of an anti-angiogenic therapeutic strategy for cervical cancer.

## Supporting Information

Figure S1 ***In vitro*** **effects of 5-aza-2′-deoxycytidine treatment and miR-196b over-expression. A)** qRT-PCR analysis of miR-196b levels in ME-180 and SiHa cells after treatment with DMSO or 2 µM 5-aza-2′-deoxycytidine (5-aza-DCT). Expression levels were normalized to RNU44 expression, relative to cells treated with DMSO. **B)** qRT-PCR analysis of miR-196b levels in ME-180 and SiHa cells after treatment with NC or pre-miR-196b (30 nmol/L). Expression levels were normalized to RNU44 expression, relative to cells treated with NC. **C)** Cell cycle analysis performed on ME-180 (top) and SiHa cells (bottom) using flow cytometry after treatment with pre-miR-196b or NC (30 nmol/L). The data represented the mean ± SEM from 3 independent experiments. NC, pre-miR Negative Control; **P<0.01; *P* = ns (not significant).
(TIF)

Figure S2 **Effect of miR-196b on** ***in vivo*** **tumor growth.** Tumor-plus-leg diameter measurements of ME-180 tumors in SCID mice after intramuscular injection of cells transfected with pre-miR-196b or Negative Control pre-miR (60 nmol/L). The plotted data represent the mean ± SEM from 9 mice in each group.
(TIF)

Figure S3 **Effect of miR-196b on** ***in vivo*** **angiogenesis and apoptosis.** Tumours removed at 25 days post-implantation were immunostained for CD31, Ki-67, and TUNEL expression; representative photomicrographs are shown for NC *vs*. miR-196b for CD31 (top), Ki-67 (middle), and TUNEL (bottom) immuno-expression. The corresponding histograms represented the mean ± SEM scoring obtained from 6 representative regions, from 2 independent tumors. NC, pre-miR Negative Control; *P<0.05; *P* = ns (not significant).
(TIF)

Figure S4 **Tri-modal strategy for target identification.** A tri-modal strategy to elucidate targets of miR-196b in cervical cancer used a combination of: i) All predicted targets of miR-196b from five *in silico* miRNA target prediction databases (*in silico*); ii) mRNA transcripts up-regulated at least 2-fold in primary cervical cancer samples compared to normal cervix tissues [Cervical cancer (Up)]; and iii) mRNA transcripts down-regulated at least 0.5-fold at both 24 and 72 hours after transfection with 30 nmol/L of pre-miR-196b (Exp. Det.).
(TIF)

Figure S5 **Transcript levels of putative miR-196b targets.** qRT-PCR analysis of: **A)** previously described; and **B)** candidate targets of miR-196b. ME-180 cells were transfected with NC or pre-miR-196b (30 nmol/L) and transcript levels of candidate targets were measured at 24 hours post-transfection. Expression levels were normalized to GAPDH expression, relative to cells transfected with pre-miR Negative Control. The data represent the mean ± SEM from 3 independent experiments. *P<0.05.
(TIF)

Figure S6 ***In vitro*** **effects of treatment with siHOXB7, siVEGF, or pre-miR-196b. A)** qRT-PCR analysis of candidate targets of HOXB7. Cells were transfected with siNEG or siHOXB7 (30 nmol/L) and transcript levels of candidate targets were measured at 24 hours post-transfection. **B)** qRT-PCR analysis of HOXB7 (left) and VEGF (right) transcript levels after treatment with siHOXB7, siVEGF, or siNEG (30 nmol/L). **C)** Western blot analysis of HOXB7 protein levels after treatment with siNEG or siHOXB7 (30 nmol/L). **D)** VEGF protein levels as measured by ELISA, after treatment with siNEG or siVEGF (30 nmol/L). E) qRT-PCR analysis of candidate targets of HOXB7. Cells were transfected with NC or pre-miR-196b (30 nmol/L) and transcript levels of candidate targets were measured at 24 hours post-transfection. The data represent the mean ± SEM from 3 independent experiments. NC, pre-miR Negative Control; siNEG, All Stars Negative Control; *P<0.05; **P<0.01.
(TIF)

Table S1 Primer sequences used for qRT-PCR.
(DOCX)
