# Supplementary figures and images for: MicroRNA-196b Regulates the Homeobox B7-Vascular Endothelial Growth Factor Axis in Cervical Cancer
Source: PLoS One. 2013 Jul 4;8(7):e67846. doi: 10.1371/journal.pone.0067846 (PMC3701631; doi:10.1371/journal.pone.0067846)

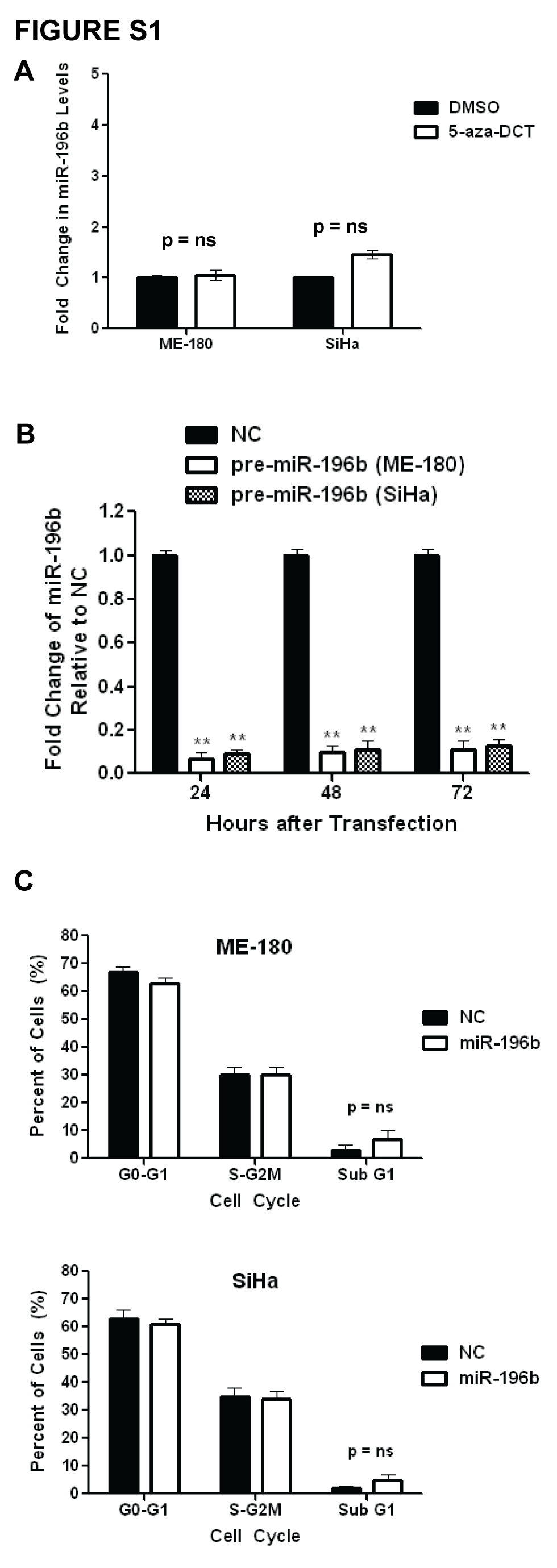

Supplement: Figure S1 — In vitro effects of 5-aza-2′-deoxycytidine treatment and miR-196b over-expression. A) qRT-PCR analysis of miR-196b levels in ME-180 and SiHa cells after treatment with DMSO or 2 µM 5-aza-2′-deoxycytidine (5-aza-DCT). Expression levels were normalized to RNU44 expression, relative to cells treated with DMSO. B) qRT-PCR analysis of miR-196b levels in ME-180 and SiHa cells after treatment with NC or pre-miR-196b (30 nmol/L). Expression levels were normalized to RNU44 expression, relative to cells treated with NC. C) Cell cycle analysis performed on ME-180 (top) and SiHa cells (bottom) using flow cytometry after treatment with pre-miR-196b or NC (30 nmol/L). The data represented the mean ± SEM from 3 independent experiments. NC, pre-miR Negative Control; **P<0.01; P = ns (not significant). (TIF) [file pone.0067846.s001.tif]

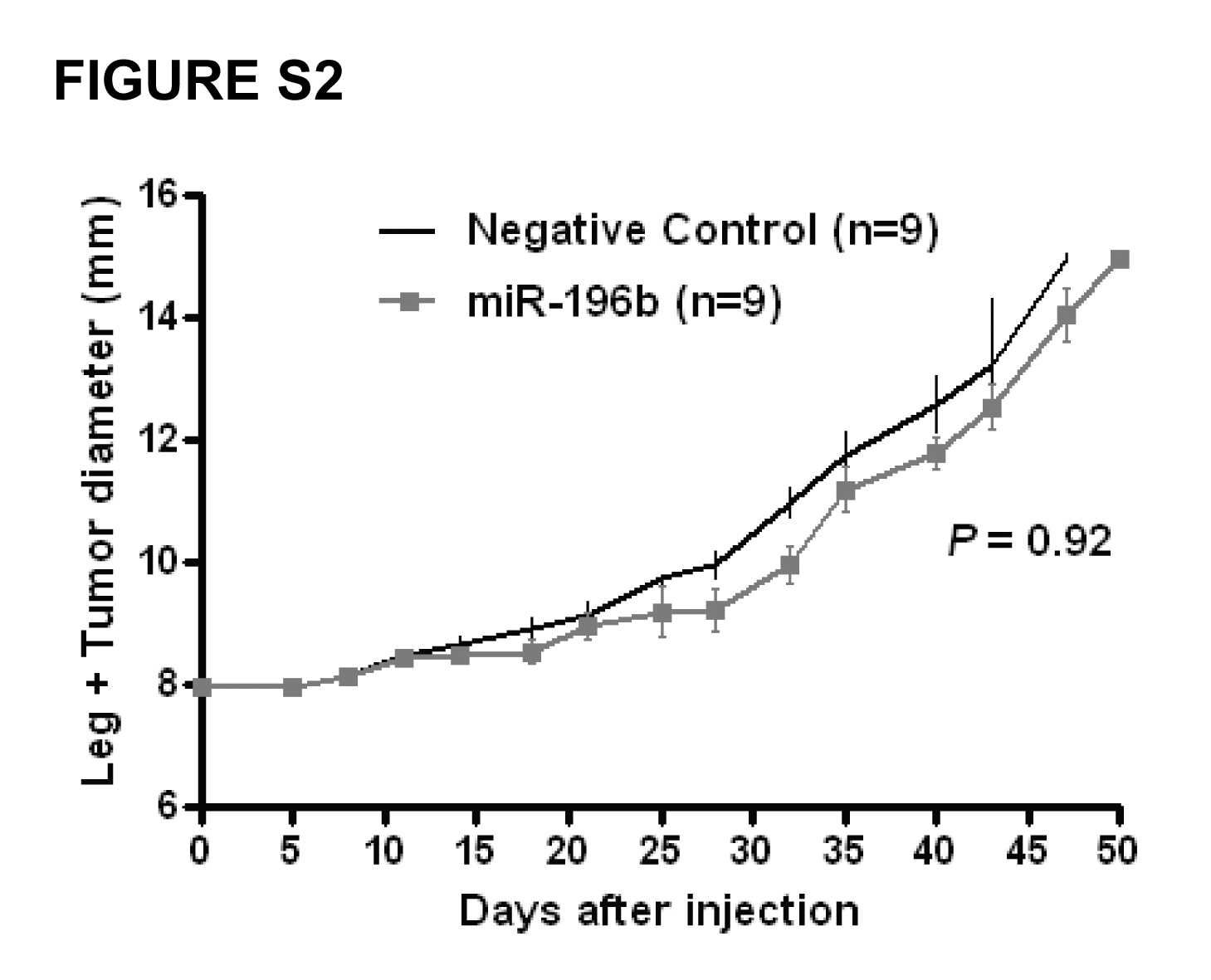

Supplement: Figure S2 — Effect of miR-196b on in vivo tumor growth. Tumor-plus-leg diameter measurements of ME-180 tumors in SCID mice after intramuscular injection of cells transfected with pre-miR-196b or Negative Control pre-miR (60 nmol/L). The plotted data represent the mean ± SEM from 9 mice in each group. (TIF) [file pone.0067846.s002.tif]

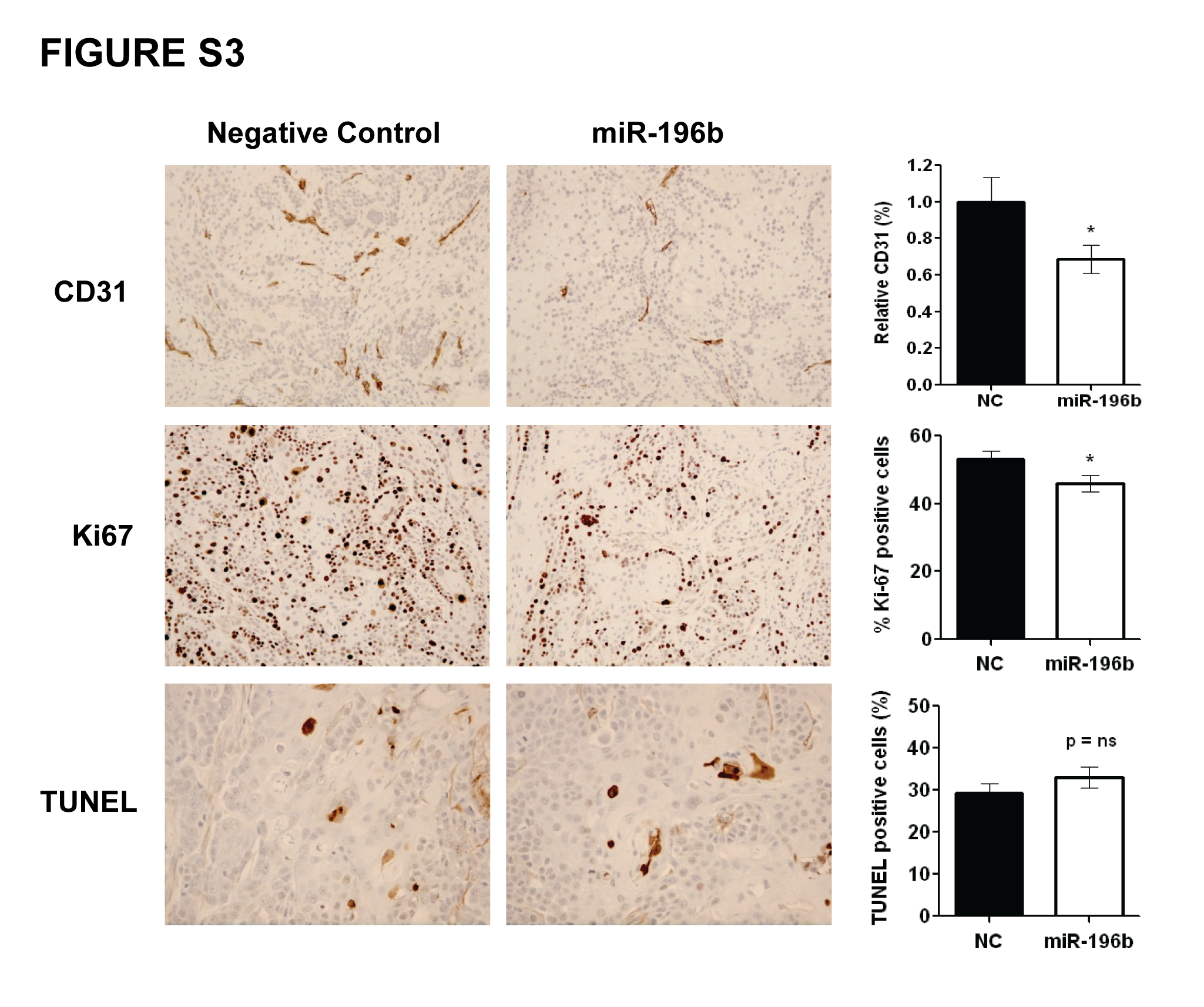

Supplement: Figure S3 — Effect of miR-196b on in vivo angiogenesis and apoptosis. Tumours removed at 25 days post-implantation were immunostained for CD31, Ki-67, and TUNEL expression; representative photomicrographs are shown for NC vs. miR-196b for CD31 (top), Ki-67 (middle), and TUNEL (bottom) immuno-expression. The corresponding histograms represented the mean ± SEM scoring obtained from 6 representative regions, from 2 independent tumors. NC, pre-miR Negative Control; *P<0.05; P = ns (not significant). (TIF) [file pone.0067846.s003.tif]

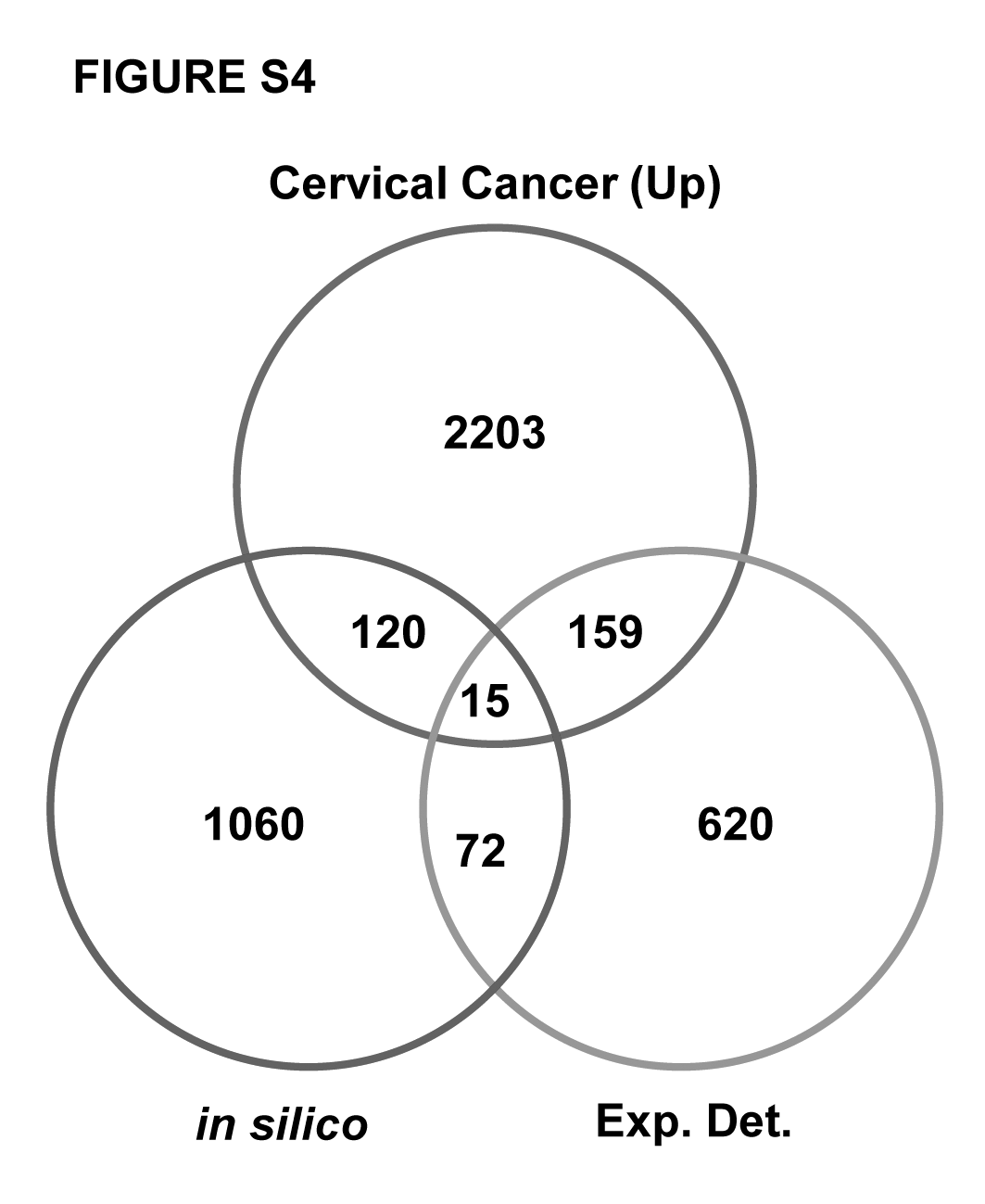

Supplement: Figure S4 — Tri-modal strategy for target identification. A tri-modal strategy to elucidate targets of miR-196b in cervical cancer used a combination of: i) All predicted targets of miR-196b from five in silico miRNA target prediction databases (in silico); ii) mRNA transcripts up-regulated at least 2-fold in primary cervical cancer samples compared to normal cervix tissues [Cervical cancer (Up)]; and iii) mRNA transcripts down-regulated at least 0.5-fold at both 24 and 72 hours after transfection with 30 nmol/L of pre-miR-196b (Exp. Det.). (TIF) [file pone.0067846.s004.tif]

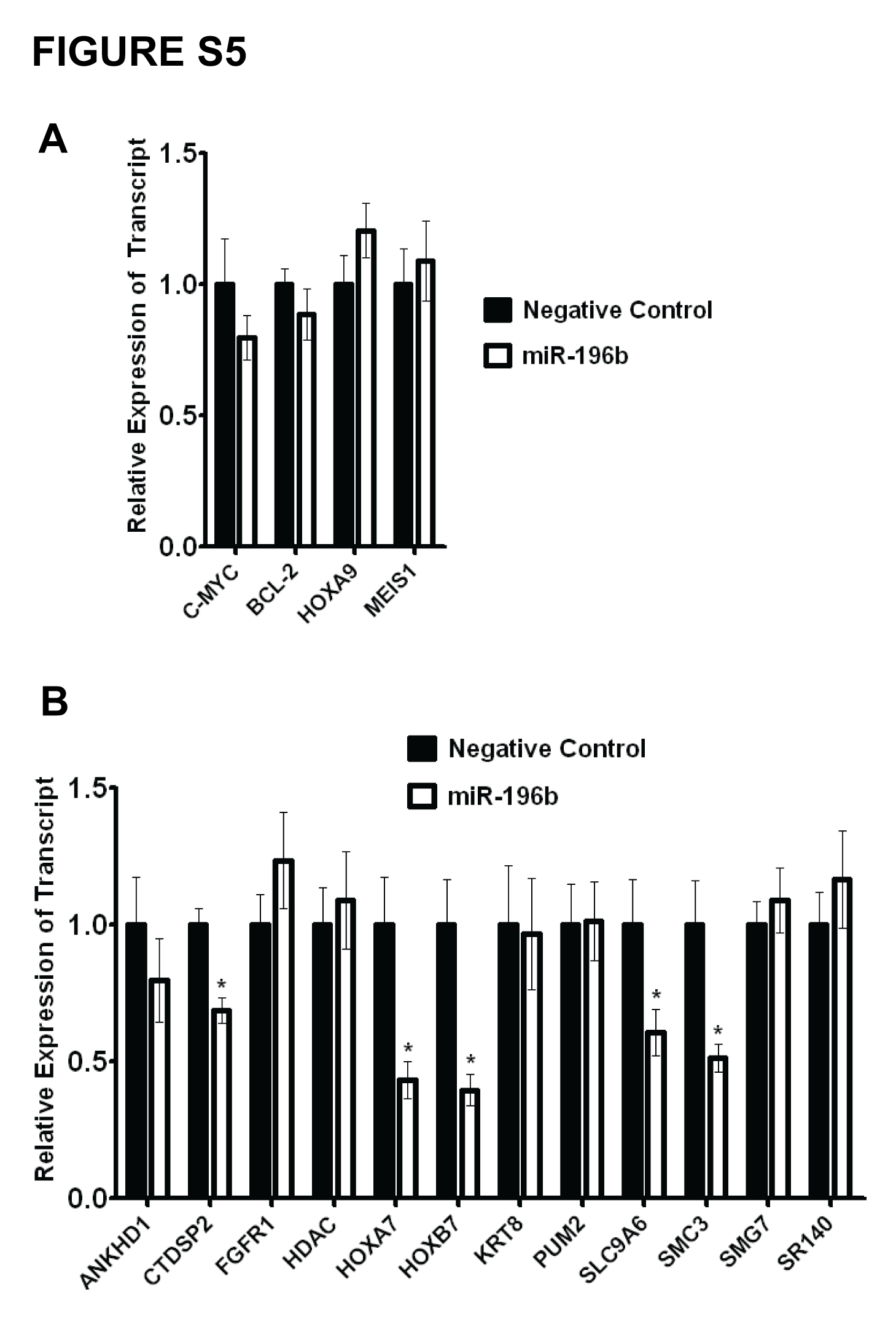

Supplement: Figure S5 — Transcript levels of putative miR-196b targets. qRT-PCR analysis of: A) previously described; and B) candidate targets of miR-196b. ME-180 cells were transfected with NC or pre-miR-196b (30 nmol/L) and transcript levels of candidate targets were measured at 24 hours post-transfection. Expression levels were normalized to GAPDH expression, relative to cells transfected with pre-miR Negative Control. The data represent the mean ± SEM from 3 independent experiments. *P<0.05. (TIF) [file pone.0067846.s005.tif]

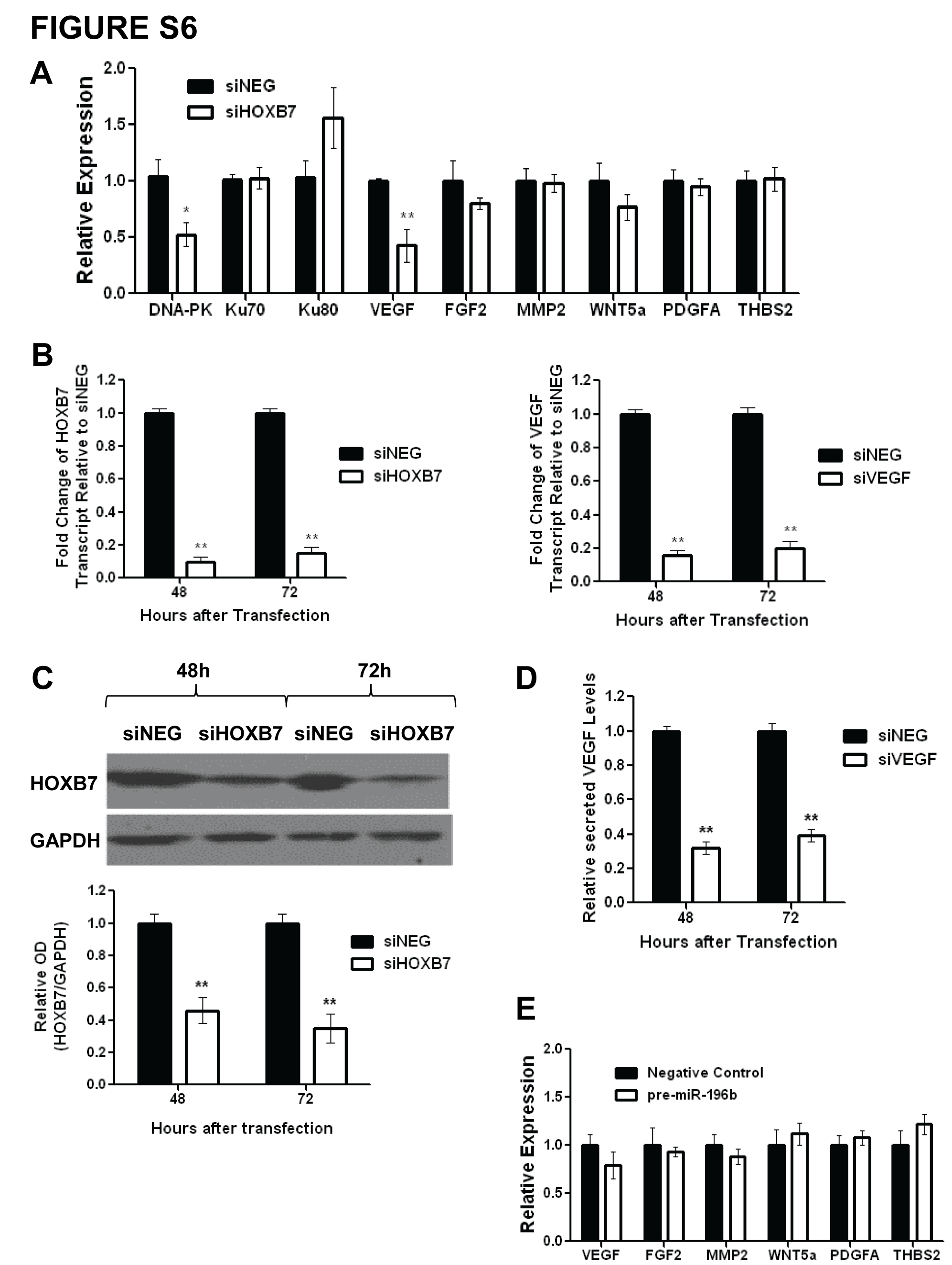

Supplement: Figure S6 — In vitro effects of treatment with siHOXB7, siVEGF, or pre-miR-196b. A) qRT-PCR analysis of candidate targets of HOXB7. Cells were transfected with siNEG or siHOXB7 (30 nmol/L) and transcript levels of candidate targets were measured at 24 hours post-transfection. B) qRT-PCR analysis of HOXB7 (left) and VEGF (right) transcript levels after treatment with siHOXB7, siVEGF, or siNEG (30 nmol/L). C) Western blot analysis of HOXB7 protein levels after treatment with siNEG or siHOXB7 (30 nmol/L). D) VEGF protein levels as measured by ELISA, after treatment with siNEG or siVEGF (30 nmol/L). E) qRT-PCR analysis of candidate targets of HOXB7. Cells were transfected with NC or pre-miR-196b (30 nmol/L) and transcript levels of candidate targets were measured at 24 hours post-transfection. The data represent the mean ± SEM from 3 independent experiments. NC, pre-miR Negative Control; siNEG, All Stars Negative Control; *P<0.05; **P<0.01. (TIF) [file pone.0067846.s006.tif]
