# Supplementary material for: MicroRNA-196b Regulates the Homeobox B7-Vascular Endothelial Growth Factor Axis in Cervical Cancer
Source: PLoS One. 2013 Jul 4;8(7):e67846. doi: 10.1371/journal.pone.0067846 (PMC3701631; doi:10.1371/journal.pone.0067846)
Supplement: Table S1 — Primer sequences used for qRT-PCR. (DOCX) [file pone.0067846.s007.docx]

**Table S1. Primer Sequences used for qRT-PCR.**

| **Gene** | **Forward Primer** | **Reverse Primer** |
| --- | --- | --- |
| HOXB7 | 5' GTTGTTACTAGTCCAGCTCTGGGAACTGAATC 3' | 5' GTTGTTAAGCTTCCACAGTCCACAAAGACAGC 3' |
| KRT8 | 5' CAAAGGCCAGAGGGCTTC 3' | 5' GACGTTCATCAGCTCCTGGT 3' |
| HDAC | 5' CCTGGACCACCAGTTCTCAC 3' | 5' CCTGTTGTTGCTTGATGTGC 3' |
| PUM2 | 5' GGGAGCTTCTCACCATTCAA 3' | 5' CCCTTCCTAAATCGAGAATCC 3' |
| SMG7 | 5' GATTTACCATGCCGTGTGAA 3' | 5' TTCTGTATCGAGCAATGTCTCC 3' |
| SR140 | 5' CGGACGGAAGATTTTCGTAT 3' | 5' CCCTCTGTTCTTCCTTAAGTGC 3' |
| ANKHD1 | 5' CGAAGTGTCCGAGGTTGAAT 3' | 5' TGCTTCTAGTCGTGCCTGTG3' |
| SMC3 | 5' AAAGAAACAGAGGGCAAACG 3' | 5' CATCAAGTTTGGCACGAGTC 3' |
| CTDSP2 | 5' AAGCAAGGCCTGGTCTCC 3' | 5' GCAATGGTGTTTGCTTCCTC 3' |
| FGFR1 | 5' CGATGTGCAGAGCATCAACT 3' | 5' AGGGGAGAGCATCTGAAACA 3' |
| HOXA7 | 5' CAGTGACCTCGCCAAAGG 3' | 5' AGGTCCTGAAGACCGCATC 3' |
| SLC9A6 | 5' CATTTGCCTTGGCCATTC 3' | 5' AATCAACACCAACCCTGATATG 3' |
| Ku70 | 5' CGACAGGTGTTTGCTGAGAA 3' | 5' CCTGGTTGGATTTTGCTTTC 3' |
| Ku80 | 5' AATCCAGGTGCAAAACGAAT 3' | 5' GGGGTTGTCTTCATTGGTGA 3' |
| DNA-PK | 5' TGCAAGGTTATAAACGCAAAA 3' | 5' ACCTCAGGAACTGTGTCAAGG 3' |
| VEGF | 5' GCTACTGCCATCCAATCGAG 3' | 5' ATCCGCATAATCTGCATGGT 3' |
| HOXA9 | 5' CCACGCTTGACACTCACACT 3' | 5' GGGTTATTGGGATCGATGG 3' |
| BCL-2 | 5' GAGGATTGTGGCCTTCTTTG 3' | 5' CATCCCAGCCTCCGTTATC 3' |
| MEIS1 | 5' TGATGGCTTGGACAACAGTG 3' | 5' AGGGTGTGTTAGATGCTGGA 3' |
| MYC | 5' TAGTGGAAAACCAGCAGCCT 3' | 5' GTTCACCATGTCTCCTCCCA 3' |
| FGF2 | 5' CTGGCTTCTAAATGTGTTACGG 3' | 5' CCCAGGTCCTGTTTTGGAT 3' |
| MMP2 | 5' TGGGCAACAAATATGAGAGAG 3' | 5' CGGCATCCAGGTTATCGGGG 3' |
| WNT5a | 5' ATTCTTGGTGGTCGCTAGGT 3' | 5' TGTACTGCATGTGGTCCTGA 3' |
| PDGFA | 5' CATGTTCTGGCCGAGGAAG 3' | 5' AGTCTATCTCCAGGAGTCGC 3' |
| THBS2 | 5' ACTCGCAGTGGAAGAACGTC 3' | 5' GAAGCAAACCCCTGAAGTGA 3' |
